# A Statistical Interaction between Circumsporozoite Protein-Specific T Cell and Antibody Responses and Risk of Clinical Malaria Episodes following Vaccination with RTS,S/AS01E

**DOI:** 10.1371/journal.pone.0052870

**Published:** 2012-12-27

**Authors:** Francis M. Ndungu, Jedidah Mwacharo, Domtila Kimani, Oscar Kai, Philippe Moris, Erik Jongert, Johan Vekemans, Ally Olotu, Philip Bejon

**Affiliations:** 1 Kenya Medical Research Institute, Centre for Geographical Medical Research (Coast), Kilifi, Kenya; 2 Nuffield Department of Clinical Medicine, John Radcliffe Hospital, University of Oxford, Oxford, United Kingdom; 3 GlaxoSmithKline Biologicals, Wavre, Belgium; 4 Centre for Clinical Vaccinology and Tropical Medicine, University of Oxford, Oxford, United Kingdom; Université Pierre et Marie Curie, France

## Abstract

The candidate malaria vaccine RTS,S/AS01_E_ provides significant but partial protection from clinical malaria. On *in vitro* circumsporozoite protein (CSP) peptide stimulation and intra-cellular cytokine staining of whole blood taken from 407 5–17 month-old children in a phase IIb trial of RTS,S/AS01_E_, we identified significantly increased frequencies of two CSP-specific CD4+ T cells phenotypes among RTS,S/AS01_E_ vaccinees (IFNγ-IL2+TNF− and IFNγ-IL2+TNF+ CD4+ T cells), and increased frequency of IFNγ-IL2-TNF+ CD4+ T cells after natural exposure. All these T cells phenotypes were individually associated with reductions in the risk of clinical malaria, but IFNγ-IL2-TNF+ CD4+ T cells independently predicted reduced risk of clinical malaria on multi-variable analysis (HR = 0.29, 95% confidence intervals 0.15–0.54, p<0.0005). Furthermore, there was a strongly significant synergistic interaction between CSP-specific IFNγ-IL2-TNF+ CD4+ T cells and anti-CSP antibodies in determining protection against clinical malaria (p = 0.002). Vaccination strategies that combine potent cellular and antibody responses may enhance protection against malaria.

## Introduction

The current lead candidate malaria vaccine is RTS,S/AS01_E_ [1]. The RTS,S antigen consists of the C-terminal region of the *P falciparum* CSP including 19 copies of the central tandem repeats, fused to the hepatitis B surface antigen (HBsAg), co-expressed with unfused HBsAg in *Saccharomyces cerevisiae* cells. The RTS,S antigen has been formulated with different adjuvants to enhance immunogenicity [2], [3]. AS01 contains the immunostimulants monophosphorly lipid A and QS21 in liposomes. RTS,S, formulated with AS01 and at a paediatric dose, is referred to as RTS,S/AS01_E_.

The vaccine induces high concentrations and frequencies of antibodies and CD4+ T cells, respectively, specific for CSP [4], [5]. Anti-CSP antibodies correlate with protection against infection in malaria-naïve-adult challenge studies [4] and field studies in young children [6], against clinical malaria in trials with young children in Kenya/Tanzania [7] and in Gabon/Ghana/Tanzania [8], but anti-CSP antibodies did not correlate with protection against clinical malaria in a trial with older children in Mozambique [9]. Anti-CSP antibodies could protect by a variety of mechanisms including complement activation, antibody dependent cellular cytotoxicity, sporozoite neutralization, and/or FcγR mediated phagocytosis [10]. CD4+ T cells might mediate protection indirectly by providing help to B cells for the production of highly effective anti-CSP Abs, or directly by secreting effector/cytotoxic cytokines (e.g. TNF or IFNγ) [11], [12].

The correlations between CD4+ T cell responses and clinical outcomes are not consistent in the literature, and this may reflect the different clinical settings (ranging from challenge studies in malaria-naïve adults [4] to Phase II field studies in African children [7]) and/or the different methods used to measure vaccine induced T cell cytokine responses (including from *ex vivo* or cultured ELISpots [13] and intracellular cytokine staining (ICS) performed on isolated PBMC [4] or ICS on whole blood assays [7], [14], [15]).

Correlations between polyfunctional T cell phenotypes and protection against malaria infection have been reported in challenge studies [4], [16], and recently, these results have been extended to identify central memory and effector/effector memory subpopulations, both of which secreted high levels of IL2, and whose frequencies were elevated in the protected relative to the unprotected groups [16].

We have previously reported our findings using a whole blood ICS assay to assess cellular responses after vaccination with RTS,S/AS01_E_ in a field trial of 447 5–17 month-old children in Kenya [7]. In that previous report, we were unable to assess polyfunctionality of T cell phenotypes, but nevertheless identified an association between the frequency of CD4+ T cells producing at least TNF on stimulation with CSP peptides and protection against clinical malaria. We have now conducted a further analysis of the flow cytometry (FACS) data using alternative software to identify polyfunctional CD4+ T cell responses, and tested for the associations of T cell phenotype with protection from clinical malaria in Kenyan children vaccinated with RTS,S/AS01_E_.

## Results

### Re-analysis and quality control

We-reanalysed the FACS acquired data, following ICS, done previously in a randomized controlled trial of the candidate malaria vaccine RTS,S/AS01_E_ in 447 5–17 month-old children in Kilifi, Kenya [7]. Samples were stained with fluorescently labelled monoclonal antibodies to IL2, TNF and IFNγ in addition to T cell markers (i.e., CD3, CD4+ and CD8+). We did not include CD40L as two previous studies using the same whole blood assay had found CD40L to be undetectable in T cells in samples taken from African children [14], [15].

From the full dataset, 6 (0.5% of 1200) samples failed quality control because of high background (>5% IFNγ+ CD4+ or CD8+ T cells on media-only control conditions), and 38 samples failed because the positive control (i.e. staphylococcal enterotoxin B (SEB) stimulation) did not result in a four-fold increase in IFNγ+ CD4+ T cells over media-only control. There were too few CD4+ T cell events acquired (<10,000) from 2 samples and too few CD8+ T cell events (<5,000) from 10 samples.

After these exclusions, data were available from 1,104 samples for CD4+ cells and 1,100 samples for CD8+ T cells. Cytokine responses were expressed as frequencies of positive cells out of 1 million CD4 or CD8 T cells. The mean background (i.e. IFNγ+ cells on media-only control) was 40 per million CD4+ T cells and 90 per million CD8+ T cells. The mean positive control results (i.e. IFNγ+ cells on SEB stimulation) were 4,000 per million CD4+ T cells and 4,000 per million CD8+ T cells.

The previous presentation of these data used FACSdiva analysis had been used to identify at least TNF+, IL2+ and IFNγ+ CD4+ T cells, without reference to polyfunctionality [7]. The present analysis with Kaluza software identified polyfunctional T cell phenotypes, but numbers of at least TNF+ and at least IL2+ CD4+ T cells could be calculated by summing all the relevant T cell phenotypes.

CD4+ T cells expressing at least TNF+ on the current Kaluza analysis correlated strongly with TNF+ cells from the previous FACSdiva analysis (Spearman's Rho = 0.88) and CD4+ T cells expressing at least IL2+ from Kaluza correlated strongly with IL2+ cells on previous FACSdiva analysis (Spearman's Rho = 0.85).

In order to quality control the analysis of polyfunctionality using Kaluza software, data from 8 volunteers were analysed twice, blind to subject labelling: the correlation coefficient between these repeated analyses was r = 0.99.

### CD4+ and CD8+ T cell cytokine responses

**Figure 1:**
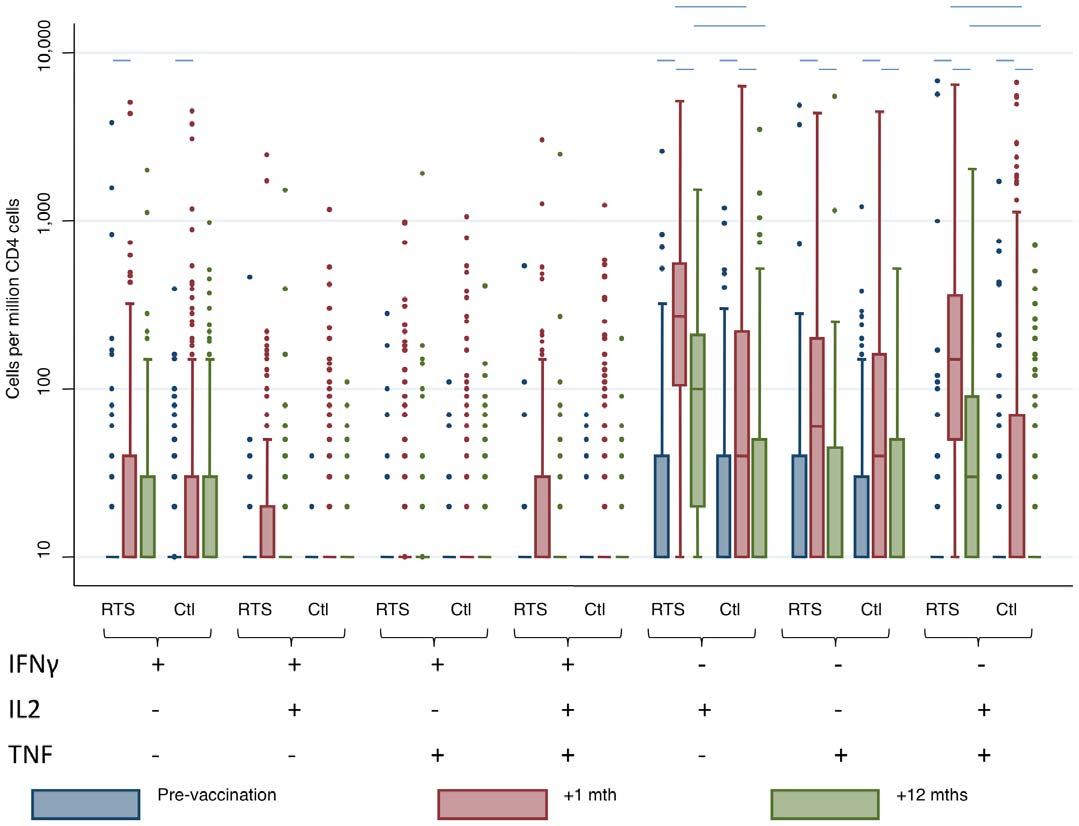
Box plots for cytokine positive T cell frequencies on stimulation with CSP peptides pre-vaccination, 1 month after the last vaccination and 12 months after the last vaccination, shown by cytokine and by vaccination group. Box plots show medians and inter-quartile ranges, the whiskers show 5^th^ to 95^th^ centiles, and outliers are shown by circles. Vaccination group is indicated by “RTS” for RTS,S/AS01_E_ and “Ctl” for rabies control. T cell phenotype is indicated by + and − for the cytokines shown far left. Significance is indicated by a horizontal line at p<0.0003 (using a Bonferroni correction for multiple comparisons).

The geometric mean frequencies of responsive CD4 and CD8 T cells (per 1 million CD4/CD8 T cells) results for all time points (i.e. pre-vaccination, 1 months after the last vaccination and 12 months after the last vaccination) and vaccination group are given in table 1, and the distributions of CD4+ T cell responses are shown in figure 1.

**Table 1:** Geometric means and 95% confidence intervals for cellular responses to CSP by vaccination group and t-test on log-transformed values for significance of difference.

Time point	RTS,S/AS01_E_ Vaccinees: Geometric Mean (95%CI)	Rabies Vaccinees: Geometric Mean (95%CI)	RTS,S,S/AS01_E_ Vaccinees: N =	Rabies Vaccinees: N =	P for T-test comparison of means
**CD4+ Cells**					
*IFNγ+ IL2-TNF−*					
Pre-vac	13 (11–15)	13 (12–15)	187	190	0.86
Vac+1	22 (18–26)	21 (17–25)	182	192	0.84
Vac+12	23 (18–29)	21 (16–27)	174	179	0.63
*IFNγ+IL2+TNF−*					
Pre-vac	10 (10–11)	10 (9–10)	187	190	0.23
Vac+1	16 (14–18)	14 (12–16)	182	192	0.28
Vac+12	14 (12–18)	11 (9–14)	174	179	0.04
*IFNγ+ IL2-TNF+*					
Pre-vac	11 (10–11)	10 (9–11)	187	190	0.18
Vac+1	16 (14–18)	15 (13–18)	182	192	0.76
Vac+12	13 (11–15)	13 (11–15)	174	179	0.72
*IFNγ+IL2+TNF+*					
Pre-vac	10 (10–11)	10 (10–11)	187	190	0.98
Vac+1	18 (15–21)	15 (13–18)	181	192	0.17
Vac+12	15 (12–20)	12 (9–15)	173	179	0.08
*IFNγ- IL2+TNF*−					
Pre-vac	22 (18–27)	22 (18–26)	187	190	0.91
Vac+1	252 (203–313)	62 (50–76)	182	192	1×10-13
Vac+12	80 (63–103)	23 (18–30)	174	179	3×10-14
*IFNγ- IL2-TNF+*					
Pre-vac	20 (17–24)	18 (15–22)	187	190	0.35
Vac+1	55 (44–70)	49 (39–61)	182	192	0.43
Vac+12	24 (19–30)	22 (18–27)	174	179	0.59
*IFNγ- IL2+TNF+*					
Pre-vac	12 (11–14)	12 (11–14)	187	190	0.86
Vac+1	142 (113–178)	31 (25–39)	182	192	9×10-16
Vac+12	36 (30–44)	14 (12–17)	174	179	5×10-13
**CD8+ Cells**					
*IFNγ+IL2-TNF*−					
Pre-vac	14 (12–17)	16 (13–18)	188	189	0.38
Vac+1	25 (20–31)	30 (24–37)	183	190	0.30
Vac+12	23 (19–28)	24 (20–30)	171	179	0.67
*IFNγ+IL2+ TNF−*					
Pre-vac	10 (9–10)	10 (9–10)	188	189	0.26
Vac+1	10 (10–10)	10 (10–10)	183	190	0.60
Vac+12	10 (9–10)	11 (10–11)	171	179	0.22
*IFNγ+IL2-TNF+*					
Pre-vac	11 (10–13)	12 (11–13)	188	189	0.83
Vac+1	15 (13–18)	18 (16–21)	183	190	0.12
Vac+12	13 (11–15)	14 (13–16)	171	179	0.36
*IFNγ+IL2+TNF+*					
Pre-vac	10 (9–10)	10 (9–10)	188	189	0.45
Vac+1	10 (10–11)	10 (9–10)	183	190	0.39
Vac+12	10 (9–12)	10 (9–12)	171	179	0.85
*IFNγ- IL2+TNF−*					
Pre-vac	30 (25–37)	29 (23–35)	188	189	0.72
Vac+1	32 (26–40)	35 (28–44)	183	190	0.56
Vac+12	52 (38–69)	36 (27–48)	171	179	0.06
*IFNγ- IL2-TNF+*					
Pre-vac	23 (19–28)	20 (17–24)	188	189	0.22
Vac+1	28 (23–34)	32 (26–39)	183	190	0.38
Vac+12	26 (21–34)	25 (20–33)	171	179	0.84
*IFNγ-TNF+IL2+*					
Pre-vac	13 (12–14)	12 (11–13)	188	189	0.17
Vac+1	15 (14–17)	15 (14–17)	183	190	0.87
Vac+12	13 (11–14)	11 (10–13)	171	179	0.05

Pre-vac = prior to vaccination, Vac+1 = 1 month after final vaccination, Vac+12 = 12 months after final vaccination.

The mean frequencies of CSP-specific cytokine positive CD8+ T cells were low, with means of 10 to 50 per million CD8+ T cells, and there were no significant differences between vaccination groups or by time-point. Therefore, data for CD8+ T cells are not described further.

### Time course of CD4+ T cell cytokine responses

In both RTS,S/AS01_E_ and control vaccinees, there were significant increases in the frequencies of CSP-specific IFNγ-IL2+TNF−, IFNγ-IL2+TNF+, and IFNγ-IL2-TNF+ CD4+ T cells during the 4 months between pre-vaccination levels and 1 month post vaccination, and a subsequent decrease in frequencies by 12 months post vaccination (Figure 1). This temporary increase in CSP specific CD4+ T cell cytokine responses among control vaccinees parallels the increase and decrease in antibodies to blood stage malaria antigens seen in the same children [17] and the timing of the malaria transmission season [17], suggesting that CSP specific CD4+ T cell responses are also acquired naturally following exposure to malaria parasites, as suggested by previous studies in adults [18], and as observed for TRAP specific CD4+ T cell responses following exposure to malaria parasites [19].

### Vaccination induced CSP-specific CD4+ T cells

Although the frequencies of CSP-specific IFNγ-IL2+TNF−, IFNγ-IL2+TNF+, and IFNγ-IL2-TNF+ CD4+ T cells increased in both vaccination groups over time, the frequencies of IFNγ-IL2+TNF− and IFNγ-IL2+TNF+ CD4+ T cells were significantly higher in the RTS,S/AS01_E_ vaccinees at 1 month (“+1”) and at 12 months (“+12”) post-vaccination (Figure 1). The frequencies of all other T cell phenotypes were similar by vaccination group.

### Correlates of protection against clinical malaria

We examined the associations between the frequencies of IFNγ-IL2+TNF−, IFNγ-IL2+TNF+, and IFNγ-IL2-TNF+ T cells and T cells expressing at least TNF or at least IL2 on stimulation with CSP and subsequent risk of clinical malaria in the 6 months that followed the measurement. Data from samples taken pre-vaccination were not included in this analysis.

**Figure 2:**
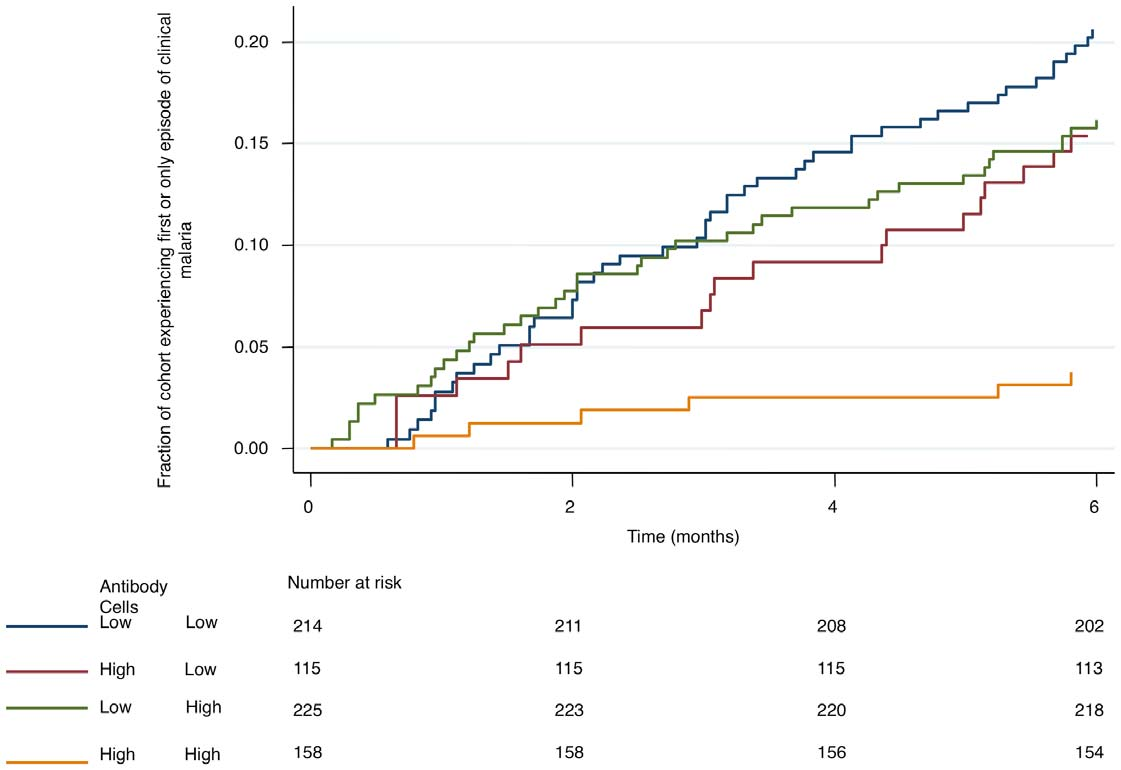
Kaplan Meier plot showing fraction of the RTS,S/AS01_E_ vaccinees experiencing an episode of clinical malaria, divided according to antibody and TNF+ only CD4+ cell anti-CSP responses. The 4 groups are as follows; 1) anti-CSP antibody titers below 40 EU/ml, TNF+ only CD4+ cells on CSP stimulation below median; 2) anti-CSP antibody titers above 40 EU/ml, TNF+ only CD4+ cells on CSP-peptide stimulation below median; 2) anti-CS antibody titers below 40 EU/ml, TNF+ CD4+ cells on CSP-peptide stimulation above median; 2) anti-CSP antibody titers above 40 EU/ml, TNF+ only CD4+ cells on CSP-peptide stimulation above median. The anti-CSP antibody titers are applied as a time-varying covariate.

We examined these associations among the RTS,S/AS01_E_ vaccinees alone, among the control vaccinees, and among both groups combined (with adjustment for vaccination group). We also examined for statistical interactions between anti-CSP antibody titres and cellular responses in determining risk of clinical malaria (Table 2). However, we could not examine these interactions among the control vaccinees, since antibody responses were detectable in less than 1% of this group, confirming earlier reports that natural exposure to malaria does not induce durable levels anti-CSP antibodies [20], [21]. The interaction between TNF+ CD4+ T cells and anti-CSP antibody titers is shown graphically in figure 2, which is based on medians, interquartile ranges, and 5^th^ to 95^th^ centiles.

**Table 2:** Hazard Ratios (HR) and 95% Confidence intervals from Cox regression models for the effect of CD4+ cellular responses to CSP on clinical malaria episodes.

		IFNγ-Il2-TNF+	IFNγ-IL2+TNF+	IFNγ-IL2+TNF−	At least TNF+	At least IL2+
*Main Effects of Cellular Responses*						
RTS,S/AS01_E_ vaccinees only.	HR	**0.29 (0.15–0.54)**	0.63 (0.40–1.00)	0.74 (0.51–1.07)	0.58 (0.35–0.95)	0.69 (0.45–1.05)
	P	**<0.0005**	0.052	0.11	0.029	0.085
Rabies vaccinees only	HR	0.77 (0.53–1.11)	0.84 (0.60–1.19)	0.95 (0.72–1.26)	0.91 (0.65–1.3)	0.78 (0.58–1.05)
	P	0.17	0.33	0.73	0.6	0.8
RTS,S/AS01E vaccinees and Rabies vaccinees pooled for analysis	HR	**0.58 (0.42–0.79)**	0.76 (0.58–0.99)	0.86 (0.69–1.07)	0.78 (0.59–1.02)	0.75 (0.59–0.94)
	P	**0.001**	0.048	0.19	0.074	0.014
*Interactions Between Cellular Responses and Antibody Levels*						
RTS,S/AS01_E_ vaccinees only	HR	0.26 (0.07–0.89)	0.41 (0.17–0.98)	0.78 (0.38–1.59)	0.54 (0.22–1.32)	0.69 (0.31–1.5)
	P	0.033	0.045	0.4	0.2	0.4
RTS,S/AS01E vaccinees and Rabies vaccinees pooled for analysis	HR	**0.22 (0.08–0.6)**	0.53 (0.29–0.98)	0.72 (0.42–1.2)	0.88 (0.66–1.17)	0.76 (0.41–1.4)
	P	**0.002**	0.042	0.2	0.4	0.4

Hazard Ratios are adjusted by age (as a continuous variable), distance from the dispensary (continuous variable), bednet use, location of residence (in 4 groupings) and, when all vaccinees are included in an analysis, by vaccination group.

“At least TNF+” refers to all cells producing TNF, including polyfunctional cells (i.e. producing TNF with IL2, TNF with IFNγ, or TNF with IFNγ and IL2). The parallel definition applies to “at least IL2+”. Results that are significant after a Bonferroni correction (i.e.<0.002) are shown in bold.

The main effects (i.e. without considering an interaction) of IFNγ-IL2+TNF−, IFNγ-IL2+TNF+, and IFNγ-IL2-TNF+ CD4+ T cells were reductions in the risk of clinical malaria of varying statistical significance. These associations were significant after a Bonferroni correction for IFNγ-IL2-TNF+ CD4+ T cells in two of the three cohorts examined (i.e. among RTS,S/AS01_E_ vaccinees, and among RTS,S/AS01_E_ and control vaccinees combined), but not among rabies control vaccinees (Table 2, significant results in bold).

The interaction between the effect of IFNγ-IL2-TNF+ CD4+ T cell frequency and anti-CSP antibodies was significant after Bonferroni correction among RTS,S/AS01_E_ vaccinees and controls combined, and significant at p = 0.033 among RTS,S/AS01_E_ vaccinees alone.

The associations between cells positive for other combinations of cytokines (i.e. IFNγ-IL2+TNF−, IFNγ-IL2+TNF+, at least IL2+ and at least TNF+ T cells) were smaller in magnitude and less significant than those between IFNγ-IL2-TNF+ CD4+ T cells and outcome, and IFNγ-IL2-TNF+ CD4+ T cells were the only significant independent cellular responses in multivariate analysis (HR = 0.57, 95%CI 0.39–0.82, p = 0.002).

There were no correlations between IFNγ-IL2-TNF+ CD4+ T cells and anti-CSP antibodies at 1 month post vaccination (correlation coefficient (r) = 0, p = 0.99) or at 6 months post vaccination (r = 0.06, p = 0.24). On the other hand, IFNγ-IL2+TNF+ and IFNγ-IL2+TNF− CD4+ T cells correlated with anti-CSP antibodies at 6 months post vaccination (r = 0.13, p = 0.02 and r = 0.15, p = 0.008, respectively), but not 1 month post vaccination (r = 0.05, p = 0.3 for both).

## Discussion

We find that vaccination of malaria-exposed children with RTS,S/AS01_E_ induces IFNγ-IL2+TNF− and IFNγ-IL2+TNF+ CD4+ T cell responses upon *in vitro* stimulation of whole blood with CSP peptides. In agreement with the results from malaria-naïve adult challenge studies [4], we find no evidence of RTS,S/AS01_E_ vaccine induced CSP-reactive CD8+ T cell responses. These findings are consistent with studies conducted in Ghana [15], where most responses were IL2+ only CD4+ T cells or IL2+TNF+ CD4+ T cells, and in Gabon [14] where responses were primarily IL2+ only CD4+ T cells with a lower IL2+TNF+ CD4+ T cell response. Although CD40L induction was seen in separated PBMC from malaria-naive adult vaccinees [4], it was not identified in either of the studies in Gabon and Ghana using the whole blood assay, and so was not measured in our study in Kenya using the whole blood assay.

We also show that IFNγ-IL2+TNF−, IFNγ-IL2+TNF+, and IFNγ-IL2-TNF+ CD4+ T cells were induced by natural exposure to malaria in the control vaccinees. The time course of acquisition and loss of these T cell phenotypes among control vaccinees closely parallels the acquisition and loss of antibody responses to blood stage parasite antigens seen our cohort [17].

Over and above the naturally acquired responses, vaccination with RTS,S/AS01_E_ induced larger and more durable CD4+ T cell cytokine responses for IFNγ-IL2+TNF− and IFNγ-IL2+TNF+ CD4+ T cells. However, vaccination with RTS,S/AS01_E_ did not significantly enhance IFNγ-IL2-TNF+ CD4+ T cell responses above those induced by natural exposure to malaria alone. This lack of IFNγ production may not be attributable to the concentration of peptides used to stimulate blood for ICS, as Olutu et al [7] used higher concentrations of the same peptides, and similarly could not detect IFNγ from ELISpot assays.

There was an association between the frequency of RTS,S/AS01_E_ induced CSP-specific CD4+ T cells and protection from clinical malaria, most strongly seen for IFNγ-IL2-TNF+ CD4+ T cells. Furthermore, there were significant interactions between CSP-specific TNF+ CD4+ T cell responses and anti-CSP antibodies induced by RTS,S/AS01_E_ vaccination. This interaction was synergistic, suggesting that the protection afforded by the combination of CD4+ T cells and anti-CSP antibodies is greater than would be predicted by their sum.

These data raise the possibility that naturally acquired cellular immunity interacts synergistically with vaccine- induced antibody-mediated immunity to enhance protection. The existence of naturally acquired cellular immunity to CSP is further supported by genetic evidence of variant specific selection pressure among T cell epitopes in CSP [22], [23], and a previously described association between CSP-peptide reactive T cells detected by cultured ELISPOT and protection against malaria in both RTS,S/AS01_E_ vaccinees and control vaccinees [13]. Furthermore, T cells responding to sporozoites may be induced by a single exposure to malaria infection [24]. On the other hand, there was no evidence for selection pressure based on CSP T epitopes in studies of RTS,S vaccinees [25], [26], and T cells responding to CSP after vaccination or natural exposure appear to be much lower in frequency than those required to demonstrate protection after vaccination with viral-vectored vaccinations [12], [27].

Our study examines associations with protection against clinical malaria and hence we must be cautious in making inferences regarding causality. Nevertheless, it is clear from studies in the field [28], [29] and in experimental challenge [4], [30] that RTS,S confers protection against asymptomatic infection and in malaria-naive volunteers, and when protection is partial this appears to result from a reduced liver to blood parasite inoculums rather than reduced blood-stage multiplication.

T cells may mediate pre-erythrocytic protection either by their direct effects on parasitized cells [31], [32], by stimulating other effector cells including natural killer cells or phagocytes [33], or may be only associated with better quality antibody responses in the absence of a causal role [34]. However, although IL2 production is associated with longer-lived anti-CSP antibodies in our study (as has been found previously, [16]), the frequency of IFNγ-IL2-TNF+ CD4+ T cells was not associated with anti-CSP antibody levels, suggesting that this is an unlikely source of confounding.

Synergistic interactions between antibody titers and magnitudes of T cell responses have been identified in mouse models of vaccination [35]. Synergy may occur because antibodies and T cells act as sequential filters, with T cells more able to protect against the reduced numbers of hepatocyte-infecting parasites that are not neutralised by antibodies. Alternatively, TNF may activate phagocytes and other innate cells like NK cells, which then act in conjunction with antibodies in mechanisms that kill and clear the opsonized pathogen [36].

Taking together the observations that TNF+ CD4+ T cells a) are induced by natural exposure to malaria (and therefore likely to vary according to transmission intensity) b) do not correlate with anti-CSP antibodies and c) interact with anti-CSP antibodies to predict outcome, it is not surprising that analyses for correlates of protection in field studies have given varying results in different cohorts [10].

In summary, we conclude that RTS,S/AS01_E_ induces CSP-specific IFNγ-IL2+TNF− and IFNγ-IL2+TNF+ CD4+ T cells. However, IFNγ-IL2-TNF+ CD4+ T cells were independently associated with protection against clinical malaria, and were induced by natural exposure. Furthermore, these T cells interacted synergistically with anti-CSP antibody to afford greater protection than either immunological response alone. These findings suggest that vaccination strategies that induce stronger cellular and antibody responses will lead to enhanced protection in the field.

## Methods

The study protocol and its amendments received ethical and scientific approval from Kenya Medical Research Institute National Ethics Committee, National Institute for Medical Research of Tanzania, the Oxford Tropical Research Ethics Committee, the London School of Hygiene and Tropical Medicine Ethics committee and the Western Institutional Review Board in Seattle. The study was conducted in accordance with the Helsinki Declaration of 1964 (revised 1996) and Good Clinical Practice guidelines and was overseen by an Independent data monitoring committee and local safety monitors. Written informed consent was obtained from parents/guardians using approved Swahili or Giriama consent forms. Illiterate parents thumb printed the consent form, which was then countersigned by an independent, literate witness.

Details on randomization, immunization and surveillance have been published previously [29]. With parental consent, children aged 5–17 months old were randomized to receive either RTS,S/AS01**_E_** or rabies vaccine in a 1∶1 ratio according to 0, 1, 2 month schedule. The primary end point was clinical malaria, defined as the presence of fever (axillary temperature ≥37.5°C) and *P. falciparum* parasitaemia ≥2500/µL. Active and passive surveillance for malaria was conducted by field workers and study personnel at local dispensaries.

Children were vaccinated between March and August 2007. Blood was taken for immunological studies before vaccination, one month post dose 3, then on March 2008 irrespective of the time of recruitment (i.e. between 4 and 10 months post dose 3, mean 8 months), 12 months post dose 3 and in October 2008 irrespective of time of recruitment, (i.e. between 12 and 18 months post dose 3, mean 15 months). Peak malaria transmission was between May and August 2008.

### CSP antibody measurement

Antibodies to the *P. falciparum* CSP tandem repeat epitope were assessed by ELISA at the Center for Vaccinology, Ghent University Hospital, Belgium. Results were reported in EU/mL. Plates were adsorbed with the recombinant antigen R32LR that contained the sequence [NVDP(NANP)15]2LR [37].

### Whole blood ICS assay

As previously published [7], whole blood was stimulated in Kilifi within 2 hours of being drawn. 350 µl of whole blood plus 100 µl of phosphate buffered saline (PBS) was incubated in three different 15 ml Falcon tube, with 1 µg/ml of anti-CD28 anti-CD4+9d monoclonal antibodies (supplied by BD). After 2 hours, Brefeldin A was added to a final concentration of 1 µg/ml and incubation was continued overnight at 37°c±1- CO_2_ 5 to 7%. EDTA was then added to a final concentration at 5 mM, and after 15 minutes 1 ml FACS lysing solution (BD). The positive control was stimulated using Staphylococcal Enterotoxin B (SEB) and negative control was PBS without peptides (media control). CSP antigen peptides were added to the third tube to a final concentration of 1 µg/ml. A set of 32 15-mer, peptides were used, overlapping by 11 amino acids to cover the full length of the CS antigen used in the vaccine (3D7 strain).

The cells were fixed and permeabilized using the Cytofix/Cytoperm buffer kit (Pharmingen). Cells were then washed in PBS and re-suspended in PBS with 10% DMSO and stored at −70°c for transport to GSK in Rixensart. In GSK, cells were thawed, washed and stained with alexa-fluor 700 conjugated anti-CD3 (Pharmingen), peridinin-chlorophyll (PerCP)-conjugated anti-CD4+ (BD Biosciences) and allophycocyanin (APC)-H7 conjugated anti-CD8+ antibodies (BD Biosciences). For intracellular staining, cells were incubated with APC conjugated anti-IL2 (Pharmingen), fluorescein-isothiocyanate (FITC)-conjugated anti-IFNγ (Pharmingen) and phycoerythrin (PE) cyanin-7 (Cy7)-conjugated anti-TNF (Pharmingen). Cells were washed, re-suspended in fetal-calf-serum (FCS)-containing phosphate buffered saline (PBS) and analyzed on a BD™ LSR II flow cytometer (BD Biosciences). We required at least 10,000 CD4++ events and 5,000 CD8++ events. Acquisition was stopped when 75,000 CD4++ events had been acquired, and we acquired more than 50,000 CD4++ events for the majority of samples (>90%). Results are expressed as numbers of cytokine positive cells per million CD4 T cells.

### Re-analysis of raw FACS data

**Figure 3:**
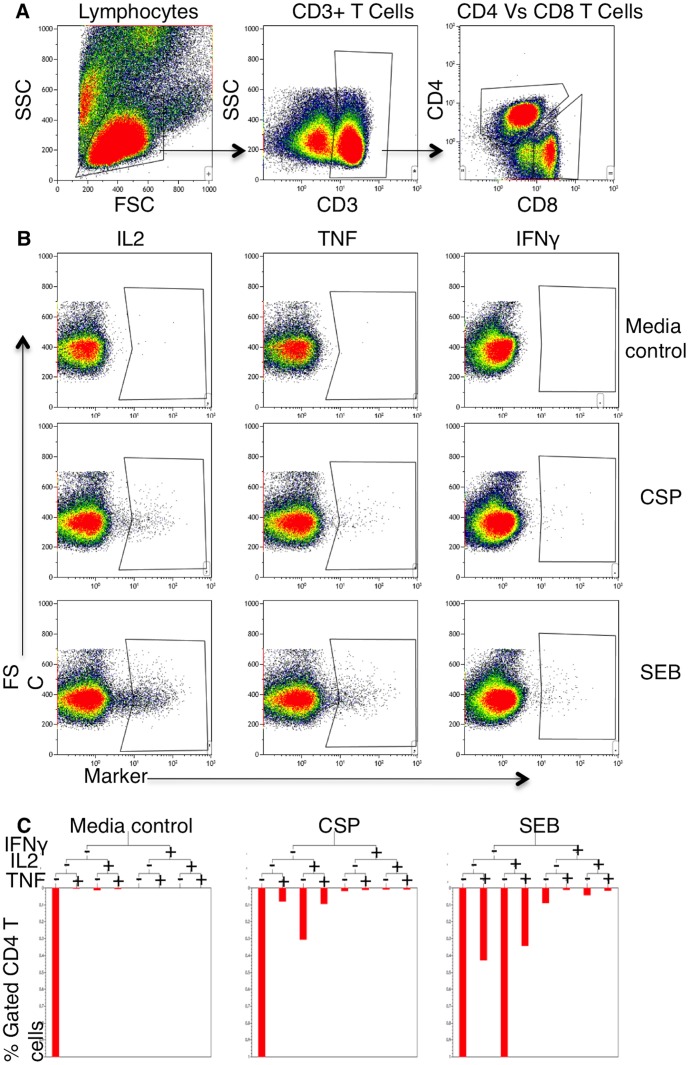
A representative example of the identification and quantitation of RTS,S/AS01_E_ induced CSP-peptide reactive CD4+ T cells producing various cytokines. A) CD4+/CD8+ T cells were gated off the side scatter (SCC) Vs CD3 gate, after gating on lymphocytes on the SSC vs forward scatter (FSC) density plot. B) Determination of the percentages of IL2+, TNF+ and IFNγ CD4+ T cells following *in vitro* stimulations with nothing (Media control), CSP and SEB. C) An example of a typical gating tree showing the identification and quantitation of CD4+ T cells producing various combinations of IL2, TNF and IFNγ, following stimulation with nothing (media control), CSP or SEB, is shown. The resultant data was expressed as percentages of cytokine positive CD4+ T cells.

An automated batch analysis of these data has already been published using the FACSDiva software (BD Biosciences) [7]. We have re-analysed the raw FACS acquired data using Kaluza software (Beckman Coulter) in order to determine polyfunctionality. Data from each individual vaccinee was analysed manually. Briefly, the pre-vaccination data was used to set up the analysis protocol for each sample. Analyses for cytokine production were done for CD3+CD4++, and for CD3+CD8++ Lymphocytes (Figure 3). A gating tree was used to hierarchically identify unique functional subsets of CD4+ T cells based on their of CD4+, IL2, TNF and IFNγ.

### Statistical analysis

Geometric mean responses are calculated and a Student's T test was performed on log-transformed values to compare between vaccination groups. Cox regression for the primary endpoint (clinical malaria with *P. falciparum* density ≥2500/µL) was adjusted for age at first vaccination, village, distance from the health facility, bed net use and anti-CSP antibody levels. Anti-CSP levels were included by dichotomizing concurrent anti-CS titers at 40 EU/mL, since this had been found to most closely correlate with protection in previous analyses [38]. Cellular responses were analyzed as time-varying covariates, applying the result from the time of the most recent clinic visit. Responses were log transformed to produce normal distributions before inclusion in the Cox regression models. The significance of interactions between cellular and antibody responses was assessed using the log-likelihood ratio test. STATA version 10 was used.
